# 

**Published:** 1904-10-15

**Authors:** 


### CONTENTS.

President's Address, - *By Charles C. Noble, D.D.S.* 487
Illogical Terminology, *By E. T. Loeffler, B.S., D.D.S.* 490
The Value of Models in Orthodontia, *By Milton T. Watson, D.D.S.* 496
Impressions in Orthodontia, - *By B. Abell, D.D.S.* 499
"Mary," - - - - *By Dr. R. E. Gilson.* 504
Address, - - - - *By Dr. Charles Godon.* 509
Prosthetic Porcelain, - *By N. S. Jenkins, D.D.S.* 512
Is There a Social Prejudice Against Dentists? - - - 515
The Use of Adrenalin Chlorid in Local Anesthesia, *By B. B. Atchison, D.D.S.* 517
Pyorrhea Alveolaris—A Case in Practice, *By H. C. Fitzhardinge, D.D.S.* 518
Editorial, - - - - - - - - 520
Announcements, - - - - - - - - 522
Bibliography, - - - - - - - - 523
Indents, - - - - - - - - - 525

